# Genetics of Growth Disorders—Which Patients Require Genetic Testing?

**DOI:** 10.3389/fendo.2019.00602

**Published:** 2019-09-06

**Authors:** Jesús Argente, Katrina Tatton-Brown, Dagmar Lehwalder, Roland Pfäffle

**Affiliations:** ^1^Hospital Infantil Universitario Niño Jesús, Universidad Autónoma de Madrid, CIBER de Fisiopatología de la Obesidad y Nutrición, Instituto de Salud Carlos III and IMDEA Institute, Madrid, Spain; ^2^Institute of Cancer Research, St George's University Hospital NHS Foundation Trust, London and St George's University of London, London, United Kingdom; ^3^Global Medical Affairs, Merck Healthcare KGaA, Darmstadt, Germany; ^4^Department of Pediatrics, University of Leipzig, Leipzig, Germany

**Keywords:** growth hormone, genetics, short stature, overgrowth, diagnosis

## Abstract

The second 360° European Meeting on Growth Hormone Disorders, held in Barcelona, Spain, in June 2017, included a session entitled *Pragmatism* vs. *Curiosity in Genetic Diagnosis of Growth Disorders*, which examined current concepts of genetics and growth in the clinical setting, in terms of both growth failure and overgrowth. For patients with short stature, multiple genes have been identified that result in GH deficiency, which may be isolated or associated with additional pituitary hormone deficiencies, or in growth hormone resistance, primary insulin-like growth factor (IGF) acid-labile subunit deficiency, IGF-I deficiency, IGF-II deficiency, IGF-I resistance, and primary PAPP-A2 deficiency. While genetic causes of short stature were previously thought to primarily be associated with the GH–IGF-I axis, it is now established that multiple genetic anomalies not associated with the GH–IGF-I axis can result in short stature. A number of genetic anomalies have also been shown to be associated with overgrowth, some of which involve the GH–IGF-I axis. In patients with overgrowth in combination with an intellectual disability, two predominant gene families, the epigenetic regulator genes, and PI3K/AKT pathway genes, have now been identified. Specific processes should be followed for decisions on which patients require genetic testing and which genes should be examined for anomalies. The decision to carry out genetic testing should be directed by the clinical process, not merely for research purposes. The intention of genetic testing should be to direct the clinical options for management of the growth disorder.

## Introduction

Human linear growth continues from the embryonic stage through to adolescence and early adulthood. During the fetal stage it is mainly controlled by insulin and growth factors and is affected by maternal health, nutrition and placental function; growth failure at this stage results in a baby being born small for gestational age. During childhood, the hypothalamic-pituitary control of the synthesis and release of growth hormone (GH), and the effects of the GH–insulin-like growth factor (IGF)-I axis on tissues throughout the body, becomes increasingly important in determining height growth. The synthesis and release of gonadotropins by the pituitary, and the increases in sex steroid, GH and IGF-I concentrations, become significant throughout puberty, and the pubertal growth spurt continues until near-adult height is reached. However, normal skeletal growth and development depends on multiple factors and can be affected by numerous genetic anomalies, including many that are not associated with the GH–IGF-I axis. If abnormal growth in either direction occurs during the pediatric period, children frequently require referral to an endocrine specialist for assessment and diagnosis.

While there are clearly environmental influences on height, which may relate to the worldwide increase in average height through the twentieth century, genetic differences have a major influence on height variation (1, 2). Height, which is one of the most heritable phenotypes in humans, shows a binomial distribution, with abnormalities involving both growth failure and overgrowth. It is a polygenic trait, and studies in multiple cohorts of twins have identified ~700 common variants that captured ~60% of heritability (1–5). For 83 identified height-associated coding variants with minor allele frequencies, effects of up to 2 cm per allele were observed, which was more than 10-times the average effect of common variants (4). Results from such genome-wide association studies have indicated several new genetic candidates, although the loci of the variants involved in height are not randomly distributed; examination of genes enriched for these loci are implicated in novel pathways involved in growth, some of which contribute to various syndromes of abnormal skeletal growth (3, 6).

While such studies indicate genes with biological relevance, genetic testing of children with abnormal growth should not just involve research to identify gene anomalies associated with growth defects, but should also provide pragmatic aspects to determine the diagnostic causes. The following report is based upon presentations from a meeting, funded by Merck Healthcare Global Medical Affairs, that aimed to examine the various genetic causes of growth disorders and the treatment options. A report from the session on adherence and personalized GH treatment in the management of growth disorders will be published separately.

## Genetic Testing for Factors Associated With Short Stature

Multiple genetic anomalies have been shown to be associated with growth failure and short stature. The short stature may be either proportionate or disproportionate, severe or mild, and have pre-natal and/or post-natal origin (7, 8). While GH treatment has been approved for use in a number of different conditions, growth response varies greatly, both between conditions and within conditions, and it is important to understand the genetic background to the condition in order to help predict the response to treatments.

Genetic testing may, therefore, be carried out for practical reasons to determine the diagnosis and treatment options in patients with growth failure; however, research is also necessary to identify genetic abnormalities that are associated with specific phenotypes linked with short stature. Development of both pragmatic and research aspects have been carried out over time, and new genetic associations are being found as the technologies become more sensitive, cheaper and more widely applied.

### The GH–IGF-I Axis

**Figure 1:**
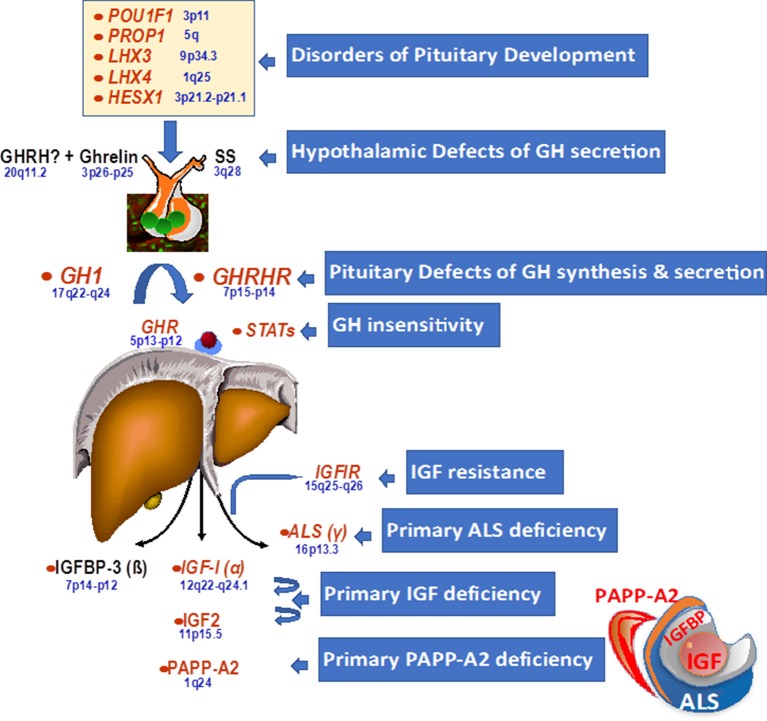
Genetic defects within the hypothalamo-pituitary axis.

Growth can be affected at various different developmental stages and at different points and facets of the GH–IGF-I axis. The genetic alterations initially identified as being associated with growth disorders are shown in Figure 1. Genetic disorders may cause defects in pituitary development during the embryonic stage, which most frequently results in multiple pituitary hormone deficiencies. There may also be abnormalities within the pituitary gland or in hypothalamic signaling, which affect GH synthesis and release by the pituitary. Finally, there may be defects in the actions of GH and IGF-I through abnormalities of receptors in target tissues, causing resistance to the effects of GH or IGF-I. These defects are usually associated with intrauterine growth retardation.

### GH Deficiency

Isolated GH deficiency or inactivity is a major cause of growth failure, and multiple genetic abnormalities have been shown over time using whole genome sequencing approaches (Table 1). In many familial cases, there are defects in, or deletions of, the gene coding for GH (*GH1*) or the GH-releasing hormone receptor (*GHRHR*), which lead to either classical GH deficiency or GH that is biologically inactive (9, 10); in cases where GH is inactive, as in Kowarski syndrome, the apparent serum concentration of GH may be normal or even slightly elevated (11). The condition may also be associated with other syndromic effects, such as ciliopathies including Alström syndrome, where genetic abnormalities are associated with functional GH deficiency (12). Ciliopathies are often associated with skeletal problems, short stature and nephronophthisis, as seen with *IFT172* mutations that cause short-rib thoracic dysplasia (13). While there are few data on GH status with ciliopathies, a case of functional GH deficiency, with low IGF-I, that responded to GH treatment has been described in a patient with an *IFT172* mutation (14).

**Table 1:** Genetic abnormalities associated with isolated GH deficiency (GHD) or bio-inactivity.

**Disorder**	**Phenotype MIM**	**Gene/locus MIM**	**Gene/locus**	**Location**	**Clinical features**	**Inheritance**	**Syndromic**
Isolated GHD, type 1A	262400	139250	*GH1*	17q23.3	No serum GH, often anti-GH antibodies	AR	
Isolated GHD, type IB	612781	139250	*GH1*	17q23.3	Low serum GH	AR	
Isolated GHD, type II	173100	139250	*GH1*	17q23.3	Variable height deficit and pituitary size; other pituitary deficits can develop	AD	
Isolated GHD, type III	307200	300300	*BTK*	Xq22.1	GHD with agammaglobulinemia	XLR	
Isolated GHD, type IV	612781	139191	*GHRHR*	7p14.3	Low serum GH	AR	
Isolated GHD, type V	618160	618016	*RNPC3*	1p21.1	Severe GHD, hypoplasia anterior pituitary	AR	
Isolated partial GHD	615925	601898	*GHSR*	3q26.31	Variable serum GH and IGF-I	AR, AD	
Kowarski syndrome (bio-inactive GH syndrome)	262650	139250	*GH1*	17q23.3	Elevated GH; deficiency of IGF-I, IGFBP-3 and acid-labile subunit	AD	
Alström syndrome	203800	606844	*ALMS1*	2p13.1	50% of cases are GH-deficient	AR	Yes
Short-rib thoracic dysplasia 10 with or without polydactyly	615630	607386	*IFT172*	2p23.3	Functional GHD, retinopathy, metaphyseal dysplasia, hypertension	AR	Yes

*AD, autosomal dominant; AR, autosomal recessive; XLR, X-linked recessive; MIM, Mendelian inheritance in man coding (https://www.ncbi.nlm.nih.gov/omim); modified from Wit et al. (9)*.

Biallelic mutations in *RNPC3* have also been associated with familial isolated GH deficiency with hypoplasia of the anterior pituitary (15). The gene encodes a structural component of the nuclear ribonucleoprotein complex that acts as a minor spliceosome in the removal of specific introns for a number of genes. The mutations were identified as being in the RNA recognition motif of the protein, which binds to U12 and acts as a bridge between U12 small nuclear RNA and U11 small nuclear RNA of the intron recognition complex. The growth failure was post-natal and proportionate, with only mild microcephaly, anterior pituitary hypoplasia, and normal psychomotor development. GH treatment of these patients was effective, despite severe short stature and late initiation of treatment (16).

Patients with isolated GH deficiency are reported to develop combined pituitary hormone deficiency in about 5–45% of cases (17, 18). For cases of combined pituitary hormone deficiencies, the genetics appear to be more complicated and the genetic etiology is frequently unidentified (Table 2). Genetic defects may occur sporadically, with no family history, or may be familial; they may be dominant where a defect in only one allele is associated with the condition, recessive where abnormalities of both alleles produce the effect, or X-linked where the defect comes from an unaffected mother and generally affects only males (19). Reported frequencies of genetic mutations in patients with combined pituitary hormone deficiencies varies greatly between countries and ethnic groups (20). For *PROP1, POU1F1*, and *HESX1*, mutation frequencies are <10% in patients with sporadic combined pituitary hormone deficiency in Western Europe, although much higher in Eastern Europe. Despite the low prevalence in Western Europe, testing for mutations of these genes is routinely carried out as part of the work-up for such patients (20).

**Table 2:** Genetic abnormalities associated with combined pituitary hormone deficiencies (CPHD).

**Disorder**	**MIM**	**Gene**	**Clinical features**	**Inheritance**	**Syndromic**
CPHD-1	613038	*POU1F1*	GH, prolactin, variable TSH deficiencies	AR, AD	
CPHD-2	262600	*PROP1*	GH, prolactin, TSH, LH, FSH, variable ACTH deficiencies; pituitary can be enlarged	AR	
CPHD-3	221750	*LHX3*	GH, prolactin, TSH, LH, FSH deficiencies; sensineural hearing loss; cervical abnormalities; short stiff neck	AR	Yes
CPHD-4	262700	*LHX4*	GH, TSH, ACTH deficiencies	AD, AR	
(CPHD-5) Septo–optic dysplasia	182230	*HESX1*	Optic nerve hypoplasia, pituitary hypoplasia, midline abnormalities of brain, absent corpus callosum, and septum pellucidum	AR, AD	Yes
CPHD-6	613986	*OTX2*	TSH, GH, LH, FSH, variable ACTH, and prolactin deficiencies	AR, AD	
Axenfeld–Rieger syndrome type 1	180500	*PITX2*	Coloboma, glaucoma, dental hypoplasia, protuberant umbilicus, brain abnormalities, variable pituitary deficiencies	AD	Yes
Optic nerve hypoplasia and CNS abnormalities	206900	*SOX2*	Variable GHD, hypogonadism, anophthalmia, developmental delay	AR	Yes
X-linked panhypopituitarism	312000, 300123	*SOX3* dup	GHD or CPHD, mental retardation	XLR	Yes
Dopa-responsive dystonia due to sepiapterin reductase deficiency	612716	*SPR*	Diurnally fluctuating movement disorder, cognitive delay, neurologic dysfunction, GH, and TSH deficiencies	AR	Yes
Holoprosencephaly	610829	*GLI2*	Holoprosencephaly, craniofacial abnormalities, polydactyly, single central incisor, partial agenesis corpus callosum (or hypopituitarism only)	AD	Yes
Pallister–Hall syndrome	146510	*GLI3*	Hypothalamic hamartoma, central polydactyly, visceral malformations	AD	Yes
IGSF1deficiency syndrome	300888	*IGSF1*	Hypothyroidism, TSH, variable GH, and prolactin deficiencies; macroorchidism	XLR	Yes
Netherton syndrome	256500	*SPINK5*	Variable GH and prolactin deficiency	AR	Yes
	600483	*FGF8*	Holoprosencephaly, septo-optic dysplasia, Moebius syndrome	AR	Yes
	136350	*FGFR1*	Hypoplasia pituitary, corpus callosum, ocular defects	AD	Yes
	607123	*PROKR2*	Variable hypopituitarism	AD	Yes
	600698	*HMGA2*	Severe GHD, ectopic posterior pituitary	AD	
	612250	*GPR161*	Pituitary stalk interruption syndrome, intellectual disability, sparse hair in frontal area, hypotelorism, broad nasal root, thick alae nasi, nail hypoplasia, short fifth finger, 2–3 toe syndactyly, hypopituitarism	AR	Yes

*AD, autosomal dominant; AR, autosomal recessive; XLR, X-linked recessive; MIM, Mendelian inheritance in man coding (https://www.ncbi.nlm.nih.gov/omim); shaded rows indicate conditions with a neurological phenotype where GH deficiency is variable; modified from Wit et al. (9)*.

Anomalies of the classic transcription factor genes cause very specific profiles of hormone deficiencies due to abnormal pituitary development; the most prevalent defects in patients with growth failure are in *PROP1*, which was reported in a literature analysis to occur in 6.7% of worldwide sporadic cases of combined pituitary hormone deficiency and 11.2% when familial cases were included (21). Mutations of *PROP1* cause the significant clinical feature of pituitary enlargement in some cases, particularly in childhood and adolescence (22), that sometimes helps in the diagnosis. Defects in genes for other transcription factors involved in pituitary development are associated with other syndromic features. For defects in *PITX, SOX2, SOX3*, and *SPR*, short stature is the predominant phenotype, while for others (shown in the shaded area of Table 2) there is a more severe neurological phenotype, and GH deficiency may or may not be present as one part of the syndrome.

Using a candidate-gene approach to identify mutations in more than 900 patients with short stature due to GH deficiency, gene defects could be identified in only about 10% of cases (23). Mutations were identified more frequently in patients with combined pituitary hormone deficiencies (14.7%) than in those with isolated GH deficiency (6.5%). In patients with combined pituitary hormone deficiencies, the most frequently identified mutations were in *PROP1* (11.6% of patients), followed by *LHX3* (1.2%), whereas the gene mutations in patients with isolated GH deficiency were most frequently found in *GH1* (4.8%), and *GHRHR* (1.1%). However, the proportion of patients with identified defects is increasing over time as more genetic variants are investigated.

Genetic abnormalities also had an effect on clinical outcomes following GH treatment (23). For 24 patients with an identified mutation and who reached near-adult height, the height standard deviation score (SDS) at start of GH treatment was −4.1, compared with a baseline height SDS of −2.9 in 191 patients without an identified mutation. The mean near-adult height SDS was −0.7 in those with a mutation vs. −0.9 in those without, and the respective mean gain in height SDS at near-adult height was 3.4 vs. 2.0, which was significantly different (*p* < 0.001). Thus, those with an identified mutation are likely to be shorter at the start of GH therapy and have a better response to GH treatment. For specific gene mutations, patients with a defect in *GH1* (*n* = 4) had an initial height SDS of −4.2 and a height SDS gain of 3.4 to reach a near-adult height SDS of −0.8. On average, these patients remained slightly shorter than normal height. For patients with a *PROP1* mutation, initial height SDS was −3.6, with a gain of 3.6 to reach height SDS 0.0 at near-adult height, and thus were normal height.

### GH Resistance and IGF-I Deficiency

The genetic abnormalities causing GH resistance or IGF-I deficiency/insensitivity (Table 3) are mainly associated with intrauterine growth retardation and being born small for gestational age. The concept of GH resistance and IGF-I deficiency is becoming more complex and cannot now be considered as a single clinical entity, but is a continuum of genetic, phenotypic, and biochemical abnormalities (24). Genetic variants influence both total IGF-I concentrations and, through changes in binding proteins, free IGF-I concentrations; therefore, it is important to determine which methodologies should be used in the diagnosis. It is often not easy to identify from the phenotype which genes should be examined, because serum levels of GH and IGFs may be decreased, normal or increased in patients with the same genetic defect. Classic examples of GH resistance are due to mutations in the GH receptor (*GHR*) gene, and a large number of missense and splice mutations and deletions in *GHR* have been identified in patients with short stature (24). Mutations in *GHR* are most generally associated with Laron syndrome, in which patients also have very low IGF-I levels (9, 11, 25). Mutations have also been reported in the signal transducer and activator of transcription gene, *STAT5B*, which is part of the signaling cascade of the GH receptor; in addition to growth failure, clinical features of these patients frequently involve immunodeficiency and pulmonary fibrosis (24, 26), although some missense mutations of *STAT5B* have recently been reported to not cause severe immune and pulmonary problems (27).

**Table 3:** Genetic abnormalities associated with GH resistance and IGF-I insensitivity.

**Syndrome**	**Phenotype MIM**	**Gene**	**Inheritance**	**Clinical characteristics**	**Laboratory findings**				
					**GH**	**GHBP**	**Prolactin**	**IGF-I**	**IGFBP-3 and ALS**
GH Resistance									
Laron syndrome	262500	*GHR*	AR (AD)	Variable height deficit, mid-facial hypoplasia	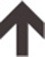	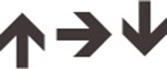		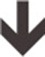	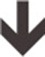
GH insensitivity with immunodeficiency	245590	*STAT5B*	AR	Mid-facial hypoplasia, immunodeficiency	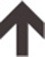	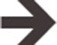	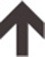	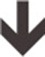	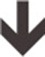
Multisystem, infantile-onset autoimmune disease	615952	*STAT3* activating	AD	Associated with early-onset multi-organ autoimmune disease					
X-linked severe combined immunodeficiency	300400	*IL2RG*	XLR	Non-responding to GH injections	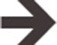	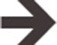		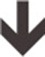	
IGF-I deficiency	608747	*IGF1*	AR	Born small for gestational age, microcephaly, deafness	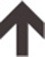	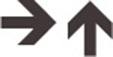		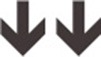	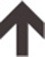
Severe growth restriction with distinctive facies	616489	*IGF2*	Patient inheritance	Short stature, facial anomalies	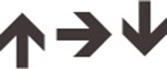			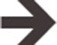	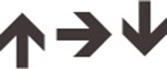
Acid labile subunit deficiency	615961	*IGFALS*	AR	Mild height deficit	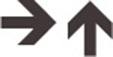	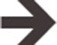		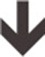	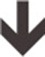
Primary protease deficiency	Not yet defined	*PAPP-A2*	AR	Moderate microcephaly, skeletal abnormalities	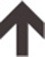			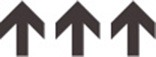	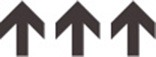
Immunodeficiency 15	615592	*IKBKB*	AR, AD	Immunodeficiency				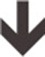	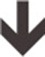
IGF insensitivity									
Resistance to IGF-I	270450	*IGF1R*	AD, AR	SGA, microcephaly	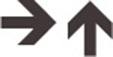			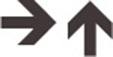	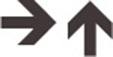

*AD, autosomal dominant; AR, autosomal recessive; XLR, X-linked recessive; MIM, Mendelian inheritance in man coding (https://www.ncbi.nlm.nih.gov/omim); arrows for laboratory findings indicate increase, decrease or no change, with more than one type of arrow indicating variability; modified from Wit et al. (9)*.

More recently, defects in *PAPP-A2* have been identified in children with progressive post-natal growth failure from two unrelated families (28, 29). The gene encodes pregnancy-associated plasma protein (PAPP)-A2, which is a metalloproteinase that cleaves insulin-like growth factor binding protein (IGFBP)-3 and IGFBP-5. Serum concentrations of total IGF-I, IGF-II, IGFBP-3, acid-labile subunit, and insulin were all increased in these patients; however, free IGF-I concentrations were very low, resulting in lack of negative feedback on GH synthesis and release. X-radiography of the fibulae, tibiae, and femurs showed that the long bones were very thin; bone density was low, with abnormal trabecular structure, and mild to moderate microcephaly was observed. After 1 year of treatment with recombinant human IGF-I, two Spanish patients showed a good response of growth velocity, height gain, and trabecular bone score (30, 31). A report has recently been published on the history and clinical implications of defects in *PAPP-A2* in human growth (32). The specific degradation of IGFBP-3 and IGFBP-5 by the active metalloproteinase results in release of free IGF-I, and it has been demonstrated that the identified mutations in the *PAPP-A2* gene, producing inactive PAPP-A2, cause increased binding of IGF-I and low bioactivity, with consequent reduced skeletal growth (33).

Identified defects resulting in GH resistance also include mutations in the *IGF1* and *IGF2* genes. Abnormalities of *IGF1* are rare in humans, with only about 9 cases reported (34, 35), although both homozygous and heterozygous mutations, and heterozygous deletions have been identified in patients with short stature (9, 36, 37); however, homozygous carriers generally have more severe growth failure than those with heterozygous defects. Homozygous *IGF1* defects that result in complete loss of functional IGF-I are associated with extreme growth failure, both pre-natal and post-natal, together with sensorineural deafness, microcephaly and mental retardation (34–38).

More recently, *IGF2* abnormalities have been detected in a number of patients; the clinical data indicated that IGF-II affects both pre-natal and post-natal growth and the genetic defects are associated with clinical features of Silver–Russell syndrome (39). While *IGF2* defects have only recently been identified, it is possible that they may be more common than initially suspected. Imprinting disorders, with hypomethylation of genetic control regions, may result in down-regulation of *IGF2* expression by the paternal allele and are involved in short stature due to both pre-natal and post-natal growth failure in Silver-Russell syndrome and Temple syndrome (40). A clinical scoring system has been derived for Silver-Russell syndrome, and a consensus guideline stated that first line molecular testing should include analysis of methylation of the H19/IGF2 domain of the 11p15 region in patients with a high likelihood of the syndrome (41). The guideline also concluded that GH treatment improved body composition and linear growth in these patients.

Defects in *IGF1R* that result in resistance to the effects of IGF-I have been described in a number of patients who had intrauterine growth retardation and failure of catch-up growth (9, 42, 43). The defects can occur in the external domain, the internal domain or in the transmembrane domain of the receptor. The condition is associated with microcephaly and psychomotor retardation in some, but not all, cases and there may be other clinical pathologies, such as impaired glucose tolerance, dysmorphic features, and cardiac abnormalities, but the pattern is inconsistent (42, 44); patients with homozygous mutations may show stronger syndromic features compared with heterozygous carriers (45). Recently, a case was described with both a deletion of *IGF1R* and a duplication of the paternal *IGF2* region, resulting in appropriate pre-natal growth but retardation of post-natal growth, indicating the key role of IGF-II in fetal growth and the more important role of the GH–IGF-I axis in post-natal growth (46). In children with *IGF1R* defects, IGF-I concentration is generally high and above normal during GH treatment, although this may be considered acceptable owing to the reduced sensitivity.

**Figure 2:**
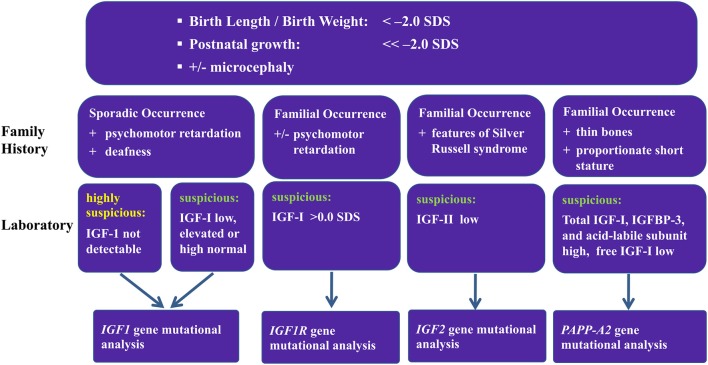
Proposed algorithm for screening patients for IGF-related genetic abnormalities.

As a result of these studies of patients with GH resistance, an algorithm was designed to try to predict which patients should have genetic screening for mutations (Figure 2). This is difficult because of the inconsistent patterns of clinical and laboratory features (Table 3). For patients with intrauterine growth retardation, possibly with microcephaly, and who have sporadic occurrence of psychomotor retardation and deafness, it may be worthwhile to look for mutations in *IGF1*; however, genotyping is somewhat ambiguous in these patients. Patients with familial occurrence of short stature related to being born small for gestational age who have high IGF-I should be checked for abnormalities of *IGF1R*. A recent study has suggested a clinical score for selecting patients for genetic testing of *IGF1R* defects, and indicated that testing should be carried out for children born small for gestational age, with low birth weight and/or length, and who remained short, had microcephaly and had IGF-I SDS >0 at presentation (47). Patients with low serum IGF-II concentration and clinical features of Silver–Russell syndrome, although not necessarily full Silver–Russell syndrome, should be examined for defects in *IGF2* (48).

In patients shown to have severe primary IGF-I deficiency, recombinant human IGF-I appears to be effective and has been approved for use (7, 49). IGF-I has also been tested in combination with GH in patients with reduced IGF-I concentrations and GH sufficiency (50). Combined GH and IGF-I treatment has also been suggested for patients with GH insufficiency plus IGF-I deficiency due to heterozygous dominant-negative *GHR* variants (51). However, IGF-I treatment has not been approved for use in such cases, and the specific clinical conditions in which GH, IGF-I, or the combination of these, may be effective need to be identified; genetic testing should help to determine which treatment approach may be useful to improve linear growth (7, 52).

For 51 patients born small for gestational age and with failure of catch-up growth, treatment with GH resulted in a median gain in height SDS of 0.64 in the first year; for nine patients born small for gestational age and without catch-up growth due to genetic defects that resulted in dysfunctional IGF-I receptors, the median first year height SDS response was 0.24 (Pfäffle R, unpublished data). The difference between the overall cohort and the patients with abnormal *IGF1R* was significant (*p* = 0.013). However, the GH dose given to the patients with dysfunctional IGF-I receptors was kept slightly lower than the dose for the patients born small for gestational age because of concerns of excessive serum IGF-I concentration, which could have partially accounted for the difference. A variable response to GH treatment of children with *IGF1R* deletions or mutations was seen in other studies, with GH in most, though not all, having a beneficial effect (44). The response in children with an *IGF1R* defect was suggested to be less than the response seen in short children born SGA due to other causes, although adult height gain was similar and may be related to duration of treatment (47). While GH treatment for children born small for gestational age and without spontaneous catch-up growth is indicated to be initiated only at age 4 years or above, starting GH treatment at a very young age (<4 years) in such children has been described to be more effective than in those who were older at GH initiation (53), at least during 3 years of therapy.

### Genetic Anomalies Not Associated With the GH–IGF-I Axis

Genome wide association studies and next-generation sequencing have indicated a large number of new genes associated with short stature, although in many cases the exact mechanism remains unknown (54). Until relatively recently, it was thought that linear growth in childhood was primarily influenced by the GH–IGF-I axis and that abnormal growth due to genetic causes were related to defects in that system. However, it has recently been suggested that rather than focusing on the GH–IGF-I axis, genetic studies should be more concerned with the biology of the growth plate (55). Cases reported as idiopathic short stature may be due to genetic disorders previously only considered to be associated with skeletal dysplasias.

Hypochondroplasia and achondroplasia are the most common skeletal dysplasias that cause short stature and are associated with abnormalities of the *FGFR3* gene (56, 57). Fibroblast growth factor receptor 3 negatively regulates chondrogenesis and mutations in the gene result in increased activity, which disrupts normal functioning of the proliferation zone of the growth plates and impairs bone elongation. While heritability is autosomal dominant, the majority of cases arise from spontaneous mutations and ~80% of cases are born to parents of average height. Diagnosis is generally at birth or in early infancy. No formal diagnostic criteria have been published although there are well-defined clinical and radiological characteristics, including rhizomelic short stature with short limbs and short fingers, limited elbow extension, small chest, macrocephaly, and flattened mid-facial features (56). GH treatment is not an approved treatment, except in Japan, but increases growth velocity over the first few years. Very few studies have followed patients to adult height, but the long-term effect appears to be limited (58). The Indian hedgehog paracrine system also controls chondrocyte proliferation and differentiation and defects can result in short stature (57, 59). Autosomal dominant mutations in the *IHH* gene result in mild skeletal dysplasias with brachydactyly, but patients may be diagnosed as born small for gestational age or as idiopathic short stature with defects only identified through whole exome sequencing, and the growth failure appeared to be improved by GH in the short-term (59).

Aggrecan is a key component of the cartilage extracellular matrix of the growth plate and articular surfaces. Abnormalities in the *ACAN* gene, which encodes the proteoglycan, results in proportionate or mildly disproportionate short stature, generally with advanced bone aging and premature growth cessation (60, 61). *ACAN* defects are a common cause of idiopathic short stature and born small for gestational age; homozygous abnormalities result in severe skeletal dysplasia while heterozygous defects cause milder skeletal dysplasia (61, 62). A clinical scoring system has been proposed to identify children likely to have an *ACAN* mutation, although there is a variable spectrum of phenotypes (63). GH treatment has been reported to be effective, but very few studies have been carried out (62, 63).

Patients with anomalies of the short stature homeobox-containing (*SHOX*) gene also show a wide spectrum of phenotypes, from Leri-Weill dischondrosteosis, with mesomelia and Madelung deformity, to non-specific short stature (64). SHOX deficiency is one of the most common genetic defects associated with short stature in humans. In patients with idiopathic short stature, the prevalence of anomalies in the *SHOX* gene or its regulatory elements, including deletions, duplications and insertions, has been reported to range from about 6 to 22%, with increasing prevalence reported as genetic tests have become more accurate (65). The severity of the condition does not appear to relate to specific gene anomalies, and individuals within a family could have the same defect but different phenotype (65, 66); however, *SHOX* deletions or mutations are more associated with Leri-Weill syndrome whereas enhancer region defects are more likely to be associated with less severe features of idiopathic short stature (67). SHOX protein expressed by the gene is a transcription factor that regulates the expression of various other genes in the growth plate, including *ACAN* and *FGFR3*; while the mechanism has not been completely elucidated, SHOX protein influences chondrocyte proliferation, hypertrophy, maturation, and apoptosis, thus regulating long bone growth (65, 68). The *SHOX* gene is located in the pseudoautosomal region and normal physiology requires two copies of the gene, with haploinsufficiency occurring when only one functioning copy of the gene is present. Short stature due to SHOX deficiency is, therefore, a frequent occurrence in children with Turner syndrome, with all or part of one X-chromosome missing. A need for screening of *SHOX* gene defects may be predicted from abnormal limb-to-trunk ratios, x-radiography and family history, and predictive algorithms have been proposed (64, 65). Where clinical features are severe, deletions are seen in about 80 to 90% and genetic evaluations should include chromosomal microarray analysis or SNP array analysis; for patients with less severe characteristics, exome sequencing is used more frequently (64, 65). GH treatment of children with SHOX deficiency is an approved indication and is effective in improving adult height by 7 to 10 cm (65, 69).

*SHOX* gene anomalies are found in ~70% of children with Leri-Weill dischondrosteosis (68). However, similar phenotypic features, including short stature but without Madelung deformity, occur with defects in the natriuretic peptide receptor-B gene, *NPR2*. NPR-B is the receptor for C-type natriuretic peptide, which is a regulator of chondrocyte differentiation and hypertrophy in the growth plates, and may be one of the genes activated by SHOX proteins (65). *NPR2* defects have been identified in about 2 to 6% of cases of idiopathic short stature (70, 71). Biallelic loss of function mutations cause Maroteaux-type acromesomelic dysplasia, while heterozygous mutations result in less severe short stature (70, 71). Therefore, patients may have severe skeletal dysplasia or non-specific skeletal abnormalities, but phenotype characteristics of patients with *NPR2* gene defects are currently not well established (72), and patients with short stature due to *NPR2* defects may only be detected where a relative has acromesomelic dysplasia (71). Very few patients with *NPR2* defects have currently been treated off-label with GH, but increased growth appeared to be modest and further studies would be required to demonstrate long-term efficacy (70, 72).

## Genetic Factors Associated With Overgrowth

### Overgrowth With No Associated Intellectual Problems

By contrast to gene defects that cause short stature, alternative defects in the same genes can result in overgrowth. Gain-of-function mutations in *NPR2* have been identified in a number of patients with skeletal overgrowth, with associated features of scoliosis and macrodactyly, with markedly large big toes (71, 73, 74). The ligand for the receptor is C-type natriuretic peptide (CNP) and overproduction due to translocation of the *NPPC* gene also caused tall stature with macrodactyly and skeletal dysplasia (75, 76). The mechanism of excess skeletal growth due to increased activation of the CNP/NRPB system was reported to be through overproduction of cyclic guanosine monophosphate within the growth plate chondrocytes (73, 74, 76).

Beckwith-Wiedemann syndrome is one of the most common overgrowth conditions and is the converse of Silver-Russell syndrome, due to molecular anomalies in the chromosome 11p15 imprinting region (47, 77, 78). Duplications of the imprinting region is associated with over-expression of the *IGF2* gene and patients are reported to have both pre-natal and post-natal overgrowth, with associated anomalies including macroglossia, organomegaly, abdominal wall defects, neonatal hypoglycemia, and predisposition to embryonic tumors (77). Because of the increased risk of tumors associated with the condition, a consensus meeting devised a scoring system based on both cardinal and suggestive clinical features of the syndrome. This allowed identification of patients requiring molecular testing and provided a flowchart for analysis of gain of methylation within the 11p15 genetic region and copy number variation (78). The guidelines also provided recommendations for management of various complications, including overgrowth that continues beyond childhood, although that occurs in only 43 to 65% of patients.

Overgrowth, with either gigantism occurring before epiphyseal growth plate fusion or acromegaly due to excessive skeletal growth at any time, can be caused by a number of genetic defects that result in benign adenomas, with excess GH and IGF-I synthesis and release. Gigantism and acromegaly are rare in children, although pediatric cases have aggressive features; identified genetic conditions include, amongst others, familial isolated pituitary adenoma and the more recently described X-linked acrogigantism (X-LAG), which have been reviewed recently and an algorithm for genetic testing and counseling proposed (79, 80). Because such diagnoses are generally made at a young age and there is frequently resistance to medical therapy, analysis of the genetic basis is needed to predict therapeutic options. The most common condition is familial isolated pituitary adenoma, which is usually diagnosed in childhood or adolescence, requires at least two cases of pituitary adenomas in the family and the tumor is large and invasive (81). Inactivating mutations or deletions of the aryl hydrocarbon receptor-interacting protein gene, *AIP*, predispose patients to the condition and are the most frequently identified genetic cause of pituitary gigantism (82). Prevalence of *AIP* mutations in cases of sporadic pituitary adenomas is reported to be ~4%, but may be up to 20% in children and adolescents with gigantism and/or macroadenomas (83). Although no genetic defects are found in a majority of patients with familial isolated pituitary adenoma (84), a recent study found novel variants of the *MEN1* gene in some cases where no defects in *AIP* were observed (85). Patients with sporadic pituitary adenomas and identified *AIP* mutations have been mainly young males, with median age of emergence at 17.5 years, the tumor tends to be larger than in patients without *AIP* mutations and tumors were more often resistant to somatostatin analogs and required surgical intervention (80, 82, 83).

X-LAG syndrome is very rare, but is the most frequently identified cause of gigantism in pre-pubertal patients, with diagnosis generally before the age of 3 years (80, 86). The condition is characterized by increased linear growth and weight that can be seen before 12 months of age, although gestational size may be normal; patients often have increased appetite and hunger and most identified cases also had hyperprolactinemia (82, 86). Because of the very early onset of overgrowth, adult height may be greatly increased at >4.5 SDS (87, 88). Both GH and IGF-I are markedly elevated, but GH releasing hormone is also elevated and the adenomas remain sensitive suggesting that therapeutic antagonists of the GH releasing hormone receptor could reduce the excess GH secretion (87, 89). The condition is linked to a germline micro-duplication of chromosome Xq26.3 and is associated with the *GPR101* gene (86–88). A number of mutations in *GPR101* have also been identified in patients with sporadic acromegaly (86, 90). *GPR101* encodes a G-protein coupled receptor, the function of which has not currently been fully elucidated, that is predominantly expressed in the hypothalamus and fetal pituitary glands, and it may have a role in pituitary ontogenesis (86, 87, 90). The disorder is X-linked, arising spontaneously in most cases; because index cases are generally female and more than 70% of cases are female, it has been suggested that affected hemizygous male embryos may be less viable, although familial cases from mother to son have been reported (79, 82, 87). X-LAG syndrome does not have any specific association with intellectual disability. However, a chromosome Xq26 microduplication was found in a 4-year old boy referred for examination for X-LAG (91). The genetic duplications are detected using array comparative genome hybridization (82, 87); this is normally clinical grade analysis, where duplications may be smaller than the assay resolution, and when assayed by high definition molecular cytogenetics the duplication did not include the *GPR101* gene (91). The patient did not show the phenotype of gigantism or GH excess and the study showed the importance of the correct genetic assay and provided supporting evidence for *GPR101* being the gene involved in X-LAG.

### Overgrowth With Intellectual Disability

Overgrowth-intellectual disability (OGID) syndromes are a family of genetic disorders characterized by excessive growth (height and/or head circumference ≥2 SD above the mean compared with the age-related peer group) in combination with an intellectual disability (92). Over the last decade, since the introduction of next-generation sequencing technologies that permit the examination of thousands of genes in a single experiment, the genetic cause of many OGID syndromes has been determined. A gene discovery study of patients with OGID syndromes, reported in 2017, included 710 participants and identified 14 different genes among 50% of affected individuals (93). Children with overgrowth phenotypes without intellectual disability were excluded from the study. Two key OGID gene families were identified: the epigenetic gene regulator family (six genes, 44% of the overall patients) and the PI3K/mTOR pathway gene family (five genes, 6% of the overall patients); in addition, variants in three genes involved in, as yet, undetermined pathways were identified (Table 4).

**Table 4:** Genetic abnormalities identified among 710 patients with OGID syndromes.

**Gene family *Variant gene***	**Proportion of total patients**	**Associated condition**	**MIM**	**Inheritance**
Epigenetic regulator genes				
*NSD1*	240 cases, 34%	Sotos syndrome	117550	AD
*EZH2*	34 cases, 4.8%	Weaver syndrome	277590	AD
*DNMT3A*	18 cases, 2.5%	Tatton-Brown–Rahman syndrome	615879	AD
*CHD8*	12 cases, 1/7%	Autism susceptibility	615032	AD
*HIST1H1E*	5 cases, 0.7%	Rahman syndrome	617537	AD
*EED*	2 cases, 0.3%	Cohen Gibson syndrome	617561	AD
PI3K/mTOR pathway genes				
*PTEN*	16 cases, 2.3%	PTEN hamartoma tumor syndrome	158350	AD
*PPP2R5D*	3 cases, 0.4%	Mental retardation	616355	AD
*MTOR*	2 cases, 0.3%	Smith-Kingsman syndrome	616638	AD
*AKT3*	1 case, 0.1%	Megalencephaly-polymicrogyria-polydactyl-hydrocephalus syndrome 2	615937	AD
*PIK3CA*	1 case, 0.1%	*PIK3CA*-related overgrowth syndrome	Multiple	Unknown
Undetermined pathway genes				
*NFIX*	14 cases, 2.0%	Marshall-Smith syndrome	602535	AD
*BRWD3*	7 cases, 1.0%	X-linked mental retardation	300659	XLR
*GPC3*	2 cases, 0.3%	Simpson-Golabi-Behmel syndrome	312870	XLR

*AD, autosomal dominant; XLR, X-linked recessive; MIM, Mendelian inheritance in man coding (http://www.omim.org); adapted from information in Tatton-Brown et al. (91)*.

The proteins encoded by genes involved in epigenetic regulation were (1) histone methyltransferases targeting specific lysine residues of histone tails (*NSD1, EED, EZH2*), (2) a DNA methyltransferase (*DNMT3A*), and (3) genes involved in chromatin remodeling (*CHD8*) and stabilization of higher-order chromatin structures (*HIST1H1E*).

Of the OGID genes identified in the study, *NSD1* was the most frequently mutated (240 children, 34% overall). Nuclear receptor binding SET domain protein 1 (NSD1) catalyzes the methylation of lysine residue 36 of histone H3 (H3K36) and, to a lesser degree, lysine residue 20 of histone H4 (H4K20), a methylation mark primarily associated with transcriptional activation (94). *NSD1* variants cause Sotos syndrome (MIM 117550), associated with increased growth, an intellectual disability and a characteristic facial appearance with a broad forehead, down-slanting palpebral fissures, long thin face, tall chin, malar flushing and sparse fronto-temporal hair (95, 96).

*EZH2* and *EED* encode components of the polycomb repressor complex 2 (PRC2), which catalyzes the methylation of histone 3 at lysine 27 (H3K27) associated with transcriptional repression (97). Pathogenic variants within *EZH2* and *EED* cause very similar OGID phenotypes; respectively, Weaver syndrome (MIM 277590) and Cohen Gibson syndrome (MIM 617561), characterized by tall stature, variable intellectual disability and characteristic facial appearance of “stuck-on” chin, horizontal skin crease, and hypertelorism (96, 98, 99).

*DNMT3A* pathogenic variants cause Tatton-Brown–Rahman overgrowth syndrome (TBRS; MIM 615879) (100). Children with TBRS characteristically have variable intellectual disability, autism spectrum disorders and increased growth (93, 100–102). A range of different medical issues are additionally associated with TBRS, including hypermobility of joints, obesity, kyphoscoliosis, and congenital cardiac anomalies (100).

*HIST1H1E* encodes the linker histone H1.4, and recently five unrelated probands were identified with *de novo* heterozygous frameshift variants (protein truncated variants, PTVs) that were clustered in a 12 base-pair region of the carboxy-terminal domain (93). Histone H1.4 is one of the 11 histones that mediate formation of chromatin structures that regulate gene expression. It is hypothesized that the clustered PTVs all generate the same open-reading frame, with the shorter mutant protein associated with a reduced net positive charge compared to the wild type, thereby disrupting normal binding to negatively-charged DNA (93, 103). Patients with *HIST1H1E* pathogenic variants have a variable intellectual disability and a characteristic appearance of high hairline and frontal bossing.

With regard to the PI3K/mTOR pathway genes, *PTEN* pathogenic variants have been an established cause of PTEN hamartoma tumor syndrome (PHTS; MIM 158350), also known for many years as Bannayan-Riley-Ruvalcaba syndrome in childhood (92, 96). The encoded PTEN protein is a negative regulator of the PI3K/mTOR pathway, and affected children are macrocephalic, and may have an intellectual disability and autistic spectrum disorder. In addition, they have a predisposition to develop cancers in adulthood (Cowden syndrome) (104). *PIK3CA* pathogenic variants are primarily associated with macrocephaly and asymmetric growth, which may be progressive (92, 96). Treatment with drugs that block the PI3K/mTOR pathway, such as sirolimus and everolimus that are already in use as anti-cancer agents, have been shown *in vitro* and in animal models to be effective in reducing the progressive overgrowth and may potentially improve cognition (93, 105, 106). Such treatments in humans have shown varying success in reducing the regional overgrowth, although the side-effect profile, most notably the increased infection rate, is significant [(106, 107), and ClinicalTrials.gov: NCT02991807 and NCT00971789].

## Conclusions

Human height follows a binomial distribution; however, individuals that lie at the extremes of the distribution may potentially have problems that require investigation. Height is a highly heritable classic polygenic trait and, therefore, part of any investigation frequently involves genetic analysis. Genome-wide studies have identified ~700 common associated variants with effects on height and 83 minor allele variants that had effects on growth of up to 2 cm per allele. Many genes have effects on the GH–IGF-I axis, and new genes are constantly being added to the list. Research is fundamental to increase knowledge of the genes and mechanisms involved in the pathophysiology of different diseases. However, in the clinical setting, genetic studies are required to progress from phenotype, auxology, and hormone measurements to more precise genetic diagnoses and treatments.

New technologies, such as whole exome sequencing and whole genome sequencing, are increasing the speed, precision and availability of genetic diagnoses, with decreasing costs (108–110). Some areas of the genome have limited capture by whole exome sequencing, making it difficult to go from a diagnosis based on phenotype and medical background to proof from genotype. Therefore, next-generation sequencing techniques, such as targeting exome sequencing, are being developed, to target specific collections of candidate genes with the use of chromosomal microarrays to detect copy number variations (16, 111). Such techniques can be directed to make a specific diagnosis, while whole exome and whole genome sequencing may be more useful for research purposes to identify new genetic variations associated with specific conditions: identifying the genes involved in combined pituitary hormone deficiency may be more useful as a scientific interest to provide new insights into pathogenesis and novel treatment strategies; identifying the genes involved in Turner syndrome will be more useful from a diagnostic point of view, when appropriate genetic counseling and treatment monitoring can be carried out; and detecting the genes involved in GH resistance could be useful for both scientific research and for identifying potential clinical consequences.

While short stature is a frequent cause of referral to a pediatric endocrinologist, screening for tall stature is generally considered a lower priority. However, it is important to identify pathological causes of gigantism and overgrowth, particularly because certain genetic conditions may be resistant to medical therapy or predispose to tumors. With regard to the OGID syndromes, while there is considerable phenotypic overlap between many of the individual conditions, patients with epigenetic regulator gene variants more frequently have tall stature in association with macrocephaly, whereas those with PI3K/mTOR pathway gene variants more usually have isolated macrocephaly. Research into the genetic basis of these syndromes provides a background understanding of the normal regulation and development of growth. On a practical basis, knowledge of the genotype in patients with OGID syndromes and in patients with growth failure is likely to be increasingly useful for pharmacogenomics and gene-directed therapies.

## Author Contributions

All authors critically revised the current work for important intellectual content and gave final approval of the version of the publication to be published. All authors have agreed to be accountable for all aspects of the work in ensuring that questions related to the accuracy or integrity of any part of it are appropriately investigated and resolved.

### Conflict of Interest Statement

DL is an employee of Merck Healthcare KGaA, Darmstadt, Germany. RP reports lecture fees from Merck, Novo Nordisk, Pfizer, and Sandoz. The remaining authors declare that the research was conducted in the absence of any commercial or financial relationships that could be construed as a potential conflict of interest.
